# Global, regional, and national trends in the disease burden and inequality of lower respiratory infections, 1990 to 2021: A systematic analysis of the 2021 global burden of disease study

**DOI:** 10.1097/MD.0000000000044280

**Published:** 2025-09-12

**Authors:** Qing-Qing Jiang, Xiao-Yu Zhang, Xiao Yu, You-De Liu, Wei Pan, Jian Xue

**Affiliations:** a Qishan Hospital of Yantai, Department of Hospital Infection Management, Shandong Province, The People’s Republic of China; b Department of Laboratory Medicine, Yantai Center for Disease Control and Prevention, Shandong Province, The People’s Republic of China; c AIDS Prevention and Control Department, Yantai Center for Disease Control and Prevention, Laishan District, Yantai, Shandong Province, People’s Republic of China.

**Keywords:** disability-adjusted life years, global burden of disease, health inequality, lower respiratory infections, socio-demographic index

## Abstract

This study analyzed the disease burden of lower respiratory infections (LRIs) and associated health inequalities globally, regionally, and nationally from 1990 to 2021, aiming to provide evidence-based insights for optimizing public health policies. Leveraging data from the 2021 global burden of diseases (BODs), injuries, and risk factors study (global burden of diseases (GBD) 2021), we comprehensively analyzed the disease burden and health inequality levels. Using frontier analysis, we aimed to elucidate the impact of national and regional development. Decomposition analysis was employed to dissect the contributions of epidemiological changes, aging, and population growth, while age-period-cohort (APC) modeling was adopted to explore temporal trends. Compared to 1990, the global burden of LRIs in 2021 exhibited a downward trend; however, significant disparities persisted across socio-demographic index (SDI) strata. In 2021, low-SDI regions recorded the highest age-standardized disability-adjusted life year (DALY) rates, with the most pronounced disease burden observed in sub-Saharan Africa. After adjusting for population size, the top 5 countries with the highest absolute disease burden were India, Nigeria, China, Pakistan, and Ethiopia, collectively accounting for 45.1% of the global burden. From 1990 to 2021, health inequalities narrowed with increasing SDI. Among all regions, China exhibited the most significant improvement, whereas the 15 countries with the largest improvement gaps were predominantly concentrated in low- and low-middle-SDI regions. Globally, reductions were primarily driven by epidemiological transitions. However, in Asia and sub-Saharan Africa, population growth substantially offset these gains. Children under 5 and adults over 65 represented the highest-burden groups. Globally, pre-2005 improvements were predominantly driven by high-SDI regions; however, post-2005, while global trends stabilized, middle-high-SDI regions exhibited a significant decline. Prior to 1947, changes were largely influenced by high-SDI regions. In contrast, over the past 3 decades (since 1987), the most significant improvements were observed in middle-high-SDI and middle-SDI regions, reflecting substantial advancements in these populations. Health inequalities related to LRIs are significantly influenced by geographic and national factors. Although epidemiological improvements have improved LRI-related health outcomes globally, these gains are offset by challenges such as population aging and growth. Targeted interventions are urgently needed to address the underlying drivers of health inequality.

## 1. Introduction

The burden of disease (BOD) encompasses the health consequences of illness, disability, and premature mortality, impacting individuals, families, and society.^[1]^ It reflects subsequent losses in population health, social wealth, and human resources, as well as the associated depletion of healthcare resources. A comprehensive assessment of BOD requires a multi-dimensional approach to evaluate its diverse implications.

Lower respiratory infections (LRIs), including acute bronchitis, influenza, pneumonia, chronic obstructive pulmonary disease, and bronchiectasis, are a major contributor to the global BOD. From 2010 to 2021,^[1]^ LRIs consistently ranked among the top 5 causes of disease burden, disproportionately affecting vulnerable populations such as children, the elderly, and those in low-income settings.^[2]^

In 2019, LRIs were responsible for an estimated 0.7 million deaths and 37.6 million disability-adjusted life years (DALYs) worldwide,^[3]^ with the COVID-19 pandemic further complicating their epidemiological burden.^[4]^ Early-life LRIs are linked to long-term respiratory sequelae, including heightened risks of chronic obstructive pulmonary disease and asthma in adulthood.^[5,6]^ Stark disparities exist in LRI mortality: low-income countries recorded rates 4 times higher than those of high-income countries, with childhood LRI fatalities exceeding 10-fold the rates observed in high-income settings^[7]^ – a gap likely driven by inequitable access to vaccines, antibiotics, and diagnostic and treatment resources.^[8,9]^ Even within middle-income and high-income countries, marginalized populations (e.g., indigenous groups and low-income communities) experience disproportionately high rates of LRI recurrence and severe outcomes due to systemic health-care disadvantages.^[10]^

Although the global burden of disease (GBD) 2021 LRIs and antimicrobial resistance collaborative group documented substantial progress in reducing LRIs mortality,^[11]^ the burden remains significant, particularly in low- and middle-income countries (LMICs). LRI mortality was associated with these infections is influenced by a range of pathogens – including influenza virus, respiratory syncytial virus (RSV), and *Streptococcus pneumoniae –* as well as risk factors such as air pollution, tobacco smoking, and malnutrition.

The DALY is a composite metric that quantifies disease burden by integrating health losses across multiple dimensions, including premature mortality and disability. It is widely used to assess inequalities in health outcomes among diverse populations and to measure the societal and economic impact of diseases, injuries, and risk factors globally. However, prior studies have not specifically examined DALYs attributable to LRIs. Thus, this study focuses on a comprehensive analysis of DALY estimates to address this gap.

This study aims to assess the impact of LRIs on global, regional, and national health inequities through a multi-dimensional lens. We will examine macro-level drivers – including national development trajectories, population growth, aging dynamics, and therapeutic advancements – to identify systemic determinants of disparity. Then, by incorporating temporal trends, we will quantify how these factors differentially shape LRI burdens across populations over time. Ultimately, our findings are intended to inform evidence-based public health strategies that address inequities in resource allocation and healthcare delivery.

## 2. Methods

### 2.1. Data source

Data were obtained from the GBD 2021 study (https://www.healthdata.org/), which compiles data from multiple sources to estimate years lived with disability, years of life lost, and DALYs for 371 diseases and injuries. LRIs, defined as pneumonia or bronchiolitis characterized by cough, fever, and shortness of breath, are the leading infectious cause of death and a major component of hospital-acquired infections. GBD modeling included cases meeting the International Classification of diseases, Ninth Revision (ICD-9) and Tenth Revision (ICD-10) diagnostic criteria for LRI (ICD-9: 079.82, 466–469, 470.0, 480–481.9, 482.0–482.89, 483.0–483.9, 484.1–484.2, 484.6–484.7, 487–490.9, 510–511.9, 513.0–513.9; ICD-10: A48.1, A70, B96.0–96.1, B97.21, B97.4–B97.6, J09–J11.89, J12–J13.9, J14–J14.0, J15–J15.8, J20–J21.9, J85.1, J91.0, P23.0–P23.4, U04–U04.9). Ill-defined codes (ICD-9: 482, 482.9–483, 484, 484.3–484.5, 484.8–486.9, 770.0, V12.61; ICD-10: J15.9, J1–J19.6, J22–J22.9, P23, P23.5–P23.9) were redistributed to LRI. Tuberculosis and COVID-19, although affecting the lower respiratory tract, were modeled separately.

### 2.2. Socio-demographic index (SDI)

The SDI, a composite measure of income, education, and fertility, was used to categorize countries into 5 SDI quintiles: low [0–0.466], low-middle [0.466–0.619], middle [0.619–0.712], high-middle [0.712–0.810], and high [0.810–1.000]. The SDI enables comparisons of health outcomes and health system performance across countries, providing insights into future health trends.

### 2.3. Statistical analysis

#### 2.3.1. DALYs

DALYs incorporate a comprehensive set of medical and sociological indicators, providing an integrated assessment of health loss from premature mortality and disability. This makes DALYs a valuable tool for identifying and analyzing health inequalities. Both DALY rates (per 100,000 population) and absolute DALY numbers were analyzed. We calculated the age-standardized DALY rate (ASDR) and its corresponding 95% confidence intervals (CIs). Temporal trends were assessed using the estimated annual percentage change (EAPC).

#### 2.3.2. Inequality analysis

The slope index of inequality (SII) and concentration index (CI) were used to measure absolute and relative inequality in LRI burden, respectively. The SII is an absolute measure of health inequality that captures the linear relationship between a health indicator and the distribution of socioeconomic status (e.g., income, education level). Specifically, it reflects the slope of the regression line linking the ASDR for LRIs to the weighted ranking of each country or region. The CI is a relative measure of health inequality that ranks populations by socioeconomic status and fits a Lorenz curve based on cumulative DALYs and cumulative population proportions. The CI ranges from −1 to 1, with values closer to 0 indicating lower inequality. A negative CI value suggests a higher BOD among populations with lower socioeconomic status.

#### 2.3.3. Frontier analysis

Frontier analysis identifies the theoretical minimum ASDR achievable for each country or territory based on its current level of development, serving as a benchmark for optimal performance. To ensure robustness, the method employs 1000 bootstrap samples to calculate the average ASDR for each SDI value, capturing the nonlinear relationship between SDI and ASDR. By measuring the absolute distance between the 2021 ASDR of each country or region and the frontier line (i.e., the efficiency gap), this approach quantifies the disparity between the current burden and the potential minimum burden, thereby identifying areas and potential for improvement.

#### 2.3.4. Decomposition analysis

This analysis quantified the contributions of aging, population growth, and epidemiological changes to changes in LRI burden.

#### 2.3.5. Age-period-cohort (APC) analysis

APC models, based on the Poisson distribution, were used to decompose temporal trends in LRI burden into age, period, and cohort effects, using relative risks (RRs).

All statistical analyses were conducted using R (version 4.2.3) and JD_GBDR (V2.24, Jingding Medical Technology Co., Ltd, Hefei, China).

## 3. Results

### 3.1. Global, regional, and national trends in the disease burden of LRIs, 1990 to 2021

#### 3.1.1. Global and national trends

**Figure 1.**
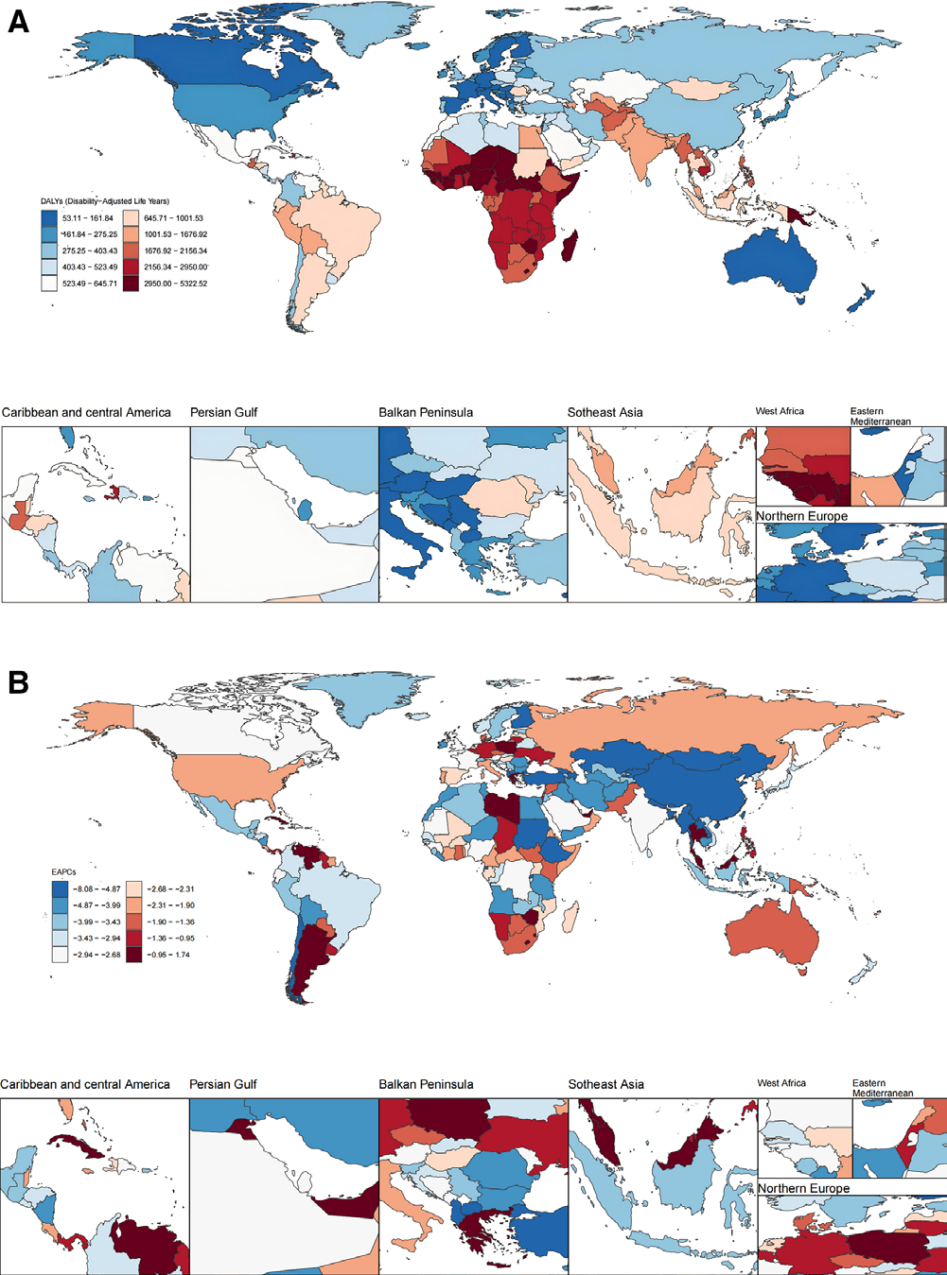
Distribution of the disease burden and inequality of LRIs in global, regional, and national, 1990–2021. (A) Age-standardized DALY rate (ASDR) in 2021. (B) Estimated annual percentage change (EAPC), 1990–2021. ASDR = age-standardized DALY rate, DALY = disability-adjusted life year, EAPC = estimated annual percentage change, LRIs = lower respiratory infections.

The global burden of LRIs decreased from 1990 to 2021, with an EAPC of −3.02 (95% CI: −3.36, −3.04). Mainland China demonstrated the largest decline (EAPC = −8.00, 95% CI: −8.34, −7.66). However, 7 countries exhibited an increasing trend: Argentina, Lesotho, Zimbabwe, Malaysia, Kuwait, Poland, and Greece (Fig. 1B). In 2021, the global LRI burden was 82,534,840.54 DALYs (95% CI: 72,611,990.16, 93,402,507.14), with an ASDR of 1168.8 (95% CI: 1016.96, 1336.94). The burden was higher in males (ASDR: 1296.46, 95% CI: 1128.84, 1490.73) than in females (ASDR: 1054.29, 95% CI: 898.51, 1194.39) (Table 1). India, Nigeria, Mainland China, Pakistan, and Ethiopia collectively accounted for 45.1% of the global LRI burden in 2021 (Fig. 1A). African countries had the highest ASDRs, followed by Asian countries (excluding East Asia) and South American countries.

**Table 1:** Epidemiological characteristics of the disease burden and inequality of lower respiratory infections in global, regional, and national, 1990–2021.

Characteristics	1990		2021		1990–2021	
Location	Numbers (millions)	ASDR (95% CI)	Numbers (millions)	ASDR (95% CI)	Case change (%)	EAPC (95% CI)
Global	204.17 (180.68–229.01)	3472.90 (3090.70–3872.10)	82.53 (72.61–93.4)	1168.80 (1016.961–1336.94)	−66.34 (−70.48, −61.50)	−3.20 (−3.35, −3.04)
SDI						
High SDI	5.03 (4.74–5.21)	525.36 (496.03–546.11)	4.33 (0.39–4.59)	217.05 (198.69–227.89)	−58.68 (−60.57, −57.27)	−2.44 (−2.64, −2.23)
High-middle SDI	13.6 (12.38–15.17)	1499.48 (1365.42–1674.59)	4.96 (4.61–5.34)	341.04 (317.96–367.57)	−77.25 (−80.23, −74.64)	−4.83 (−5.00, −4.66)
Middle SDI	53.99 (4.91–59.43)	2994.73 (2744.80–3259.17)	15.75 (14.37–17.13)	762.14 (692.26–834.15)	−74.55 (−77.28, −71.45)	−4.11 (−4.23, −3.99)
Low-middle SDI	72.43 (63.53–81.68)	4608.42 (4088.78–5139.41)	26.75 (23.18–30.51)	1575.14 (1374.89–1781.31)	−65.82 (−70.50, −60.13)	−2.98 (−3.16, −2.81)
Low SDI	59 (48.39–70.45)	7438.05 (6344.84–8574.92)	30.68 (24.88–36.96)	2580.61 (2198.67–2993.43)	−65.31 (−70.25, −59.29)	−3.18 (−3.33, −3.03)
Regions						
Andean Latin America	2.11 (1.87–2.38)	4685.05 (4234.66–5194.51)	0.73 (0.6–0.89)	1221.86 (1004.48–1485.31)	−73.92 (−78.71, −67.87)	−3.83 (−4.02, −3.65)
Australasia	0.05 (0.05–0.06)	261.90 (246.11–276.23)	0.05 (0.05–0.06)	100.72 (88.04–108.87)	−61.54 (−64.95, −58.55)	−2.25 (−2.75, −1.74)
Caribbean	0.96 (0.83–1.11)	2519.14 (2199.67–2866.39)	0.53 (0.43–0.64)	1196.22 (956.61–1461.49)	−52.52 (−62.45, −42.22)	−1.82 (−1.99, −1.65)
Central Asia	4.57 (4.27–4.89)	4997.56 (4687.41–5338.12)	1.23 (1.05–1.43)	1280.95 (1105.25–1488.76)	−74.37 (−78.11, −70.11)	−4.33 (−4.67, −3.99)
Central Europe	1.19 (1.15–1.23)	1159.48 (1114.69–1205.25)	0.66 (0.62–0.7)	399.46 (370.68–427.04)	−65.55 (−68.21, −62.89)	−3.13 (−3.46, −2.80)
Central Latin America	3.91 (3.68–4.18)	2043.83 (1942.74–2163.58)	1.54 (1.35–1.79)	671.271 (581.649–784.789)	−67.15 (−71.73, −61.41)	−3.08 (−3.39, −2.77)
Central Sub-Saharan Africa	5.9 (4.46–7.27)	7269.85 (5839.20–8711.07)	2.99 (2.39–3.7)	2860.40 (2268.24–3619.32)	−60.65 (−68.57, −48.90)	−3.02 (−3.16, −2.87)
East Asia	33.1 (28.64–38.03)	3067.74 (2674.19–3502.69)	4.42 (3.8–5.13)	355.33 (307.92–411.05)	−88.41 (−90.63, −85.82)	−7.78 (−8.10, −7.47)
Eastern Europe	1.31 (1.28–1.35)	729.19 (706.62–751.77)	0.98 (0.89–1.07)	400.28 (370.08–433.37)	−45.10 (−49.85, −39.88)	−2.07 (−2.83, −1.31)
Eastern Sub-Saharan Africa	21.61 (17.75–26.33)	7551.04 (6530.46–8817.61)	9.34 (7.75–11.04)	2471.70 (2159.57–2795.47)	−67.26 (−72.07, −61.14)	−3.577 (−3.696, −3.457)
High-income Asia Pacific	1.19 (1.11–1.24)	704.58 (649.98–737.08)	1.18 (1–1.29)	219.90 (194.44–235.11)	−68.79 (−70.62, −67.25)	−3.12 (−3.39, −2.85)
High-income North America	1.42 (1.32–1.47)	427.22 (400.11–442.85)	1.06 (0.95–1.13)	183.71 (170.07–194.81)	−56.90 (−58.55, −55.11)	−2.25 (−2.54, −1.96)
North Africa and Middle East	13.94 (12.05–17.29)	3039.24 (2659.87–3678.19)	3.7 (3.19–4.27)	709.066 (616.017–810.704)	−76.67 (−81.33, −72.79)	−4.07 (−4.19, −3.94)
Oceania	0.45 (0.37–0.55)	5021.77 (4234.68–5987.06)	0.48 (0.38–0.6)	2823.21 (2282.01–3449.90)	−43.78 (−55.09, −28.81)	−1.29 (−1.54, −1.03)
South Asia	61.4 (52.07–69.74)	4317.75 (3716.95–4854.02)	22.64 (19.52–26.05)	1507.04 (1299.12–1732.15)	−65.09 (−70.74, −57.01)	−2.89 (−3.10, −2.68)
Southeast Asia	17.36 (15.28–20.31)	3315.81 (2952.62–3811.85)	6.28 (5.51–7.06)	1079.45 (944.05–1215.35)	−67.44 (−72.27, −61.79)	−3.15 (−3.26, −3.04)
Southern Latin America	0.46 (0.45–0.48)	986.73 (953.90–1016.64)	0.61 (0.56–0.65)	738.16 (680.62–787.15)	−25.19 (−30.11, −20.27)	0.12 (−0.18, 0.42)
Southern Sub-Saharan Africa	2.51 (2.25–2.82)	4298.73 (3897.51–4732.72)	1.9 (1.65–2.15)	2664.77 (2337.03–2997.19)	−38.01 (−47.08, −29.73)	−0.87 (−1.44, −0.30)
Tropical Latin America	3.16 (2.91–3.45)	2232.53 (2074.10–2409.53)	1.72 (1.57–1.85)	723.28 (657.67–782.71)	−67.60 (−71.31, −63.87)	−2.92 (−3.25, −2.58)
Western Europe	1.81 (1.69–1.88)	357.00 (336.21–368.20)	1.56 (1.34–1.67)	154.98 (138.28–163.94)	−56.59 (−58.68, −54.94)	−2.50 (−2.85, −2.16)
Western Sub-Saharan Africa	25.75 (20.61–30.84)	8126.58 (6791.40–9537.88)	18.94 (14.18–24.26)	3258.66 (2537.04–4045.21)	−59.90 (−67.01, −52.61)	−2.59 (−2.80, −2.38)

ASDR = age-standardized DALY rate, CI = confidence interval, DALY = disability-adjusted life year, EAPC = estimated annual percentage change, SDI = socio-demographic index.

#### 3.1.2. Regional trends

**Figure 2.**
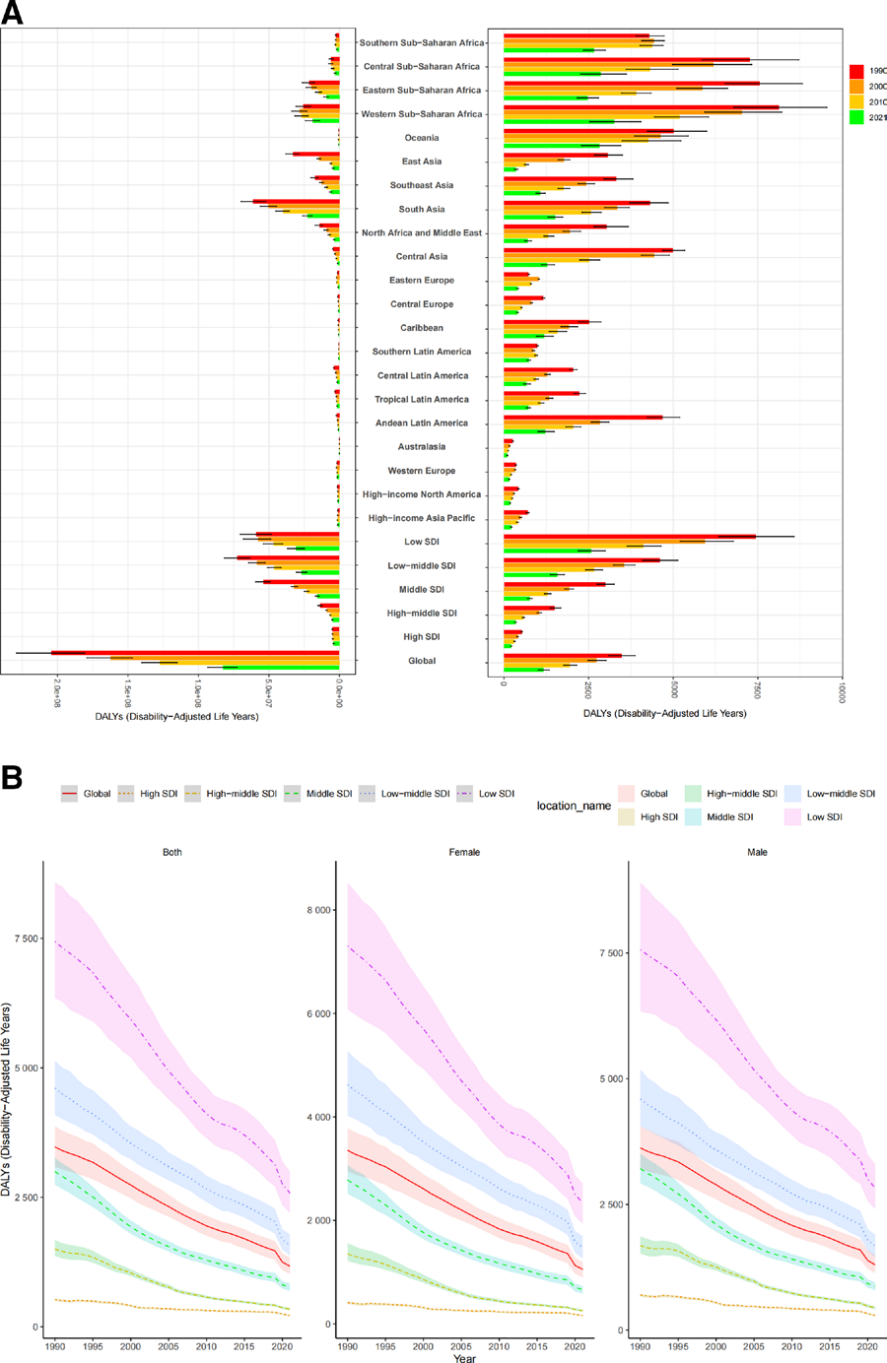
Distribution of LRI burden by SDI and region (1990–2021). (A) Trends in DALY numbers and ASDR of LRIs by region level (1990, 2000, 2010, 2021). (B) Trends in ASDR of LRIs by SDI level (1990–2021). ASDR = age-standardized DALY rate, DALY = disability-adjusted life year, LRI = lower respiratory infection, SDI = socio-demographic index.

No significant correlation was observed between the EAPC and ASDR (*R* = 0.13, *P* > .05) or between the EAPC and SDI (*R* = 0.12, *P* > .05). However, ASDR decreased significantly with increasing SDI (*R* = −0.90, *P* < .001) (Fig. 2B). Low-SDI and low-middle-SDI regions exhibited ASDRs higher than the global average (Table 1, Fig. 2B), with low-SDI countries showing the highest burden (ASDR: 2580.61, 95% CI: 2198.67, 2993.43). Eastern Europe, Southern Sub-Saharan Africa, and Southern Latin America showed minimal declines before 2010 but experienced substantial reductions thereafter.

In 2021, Australia had the lowest LRI burden, both in DALY numbers and ASDR. Southern Sub-Saharan Africa, particularly the Western region (ASDR: 3258.66, 95% CI: 2537.04, 4045.21), had the highest ASDR. When adjusted for population size, South Asia had the highest absolute DALY burden (22,635,505.04, 95% CI: 19,516,425.27, 26,049,496.29), followed by Sub-Saharan Africa (both Western and Eastern). In contrast, regions such as Andean Latin America, the Caribbean, and Oceania, despite their relatively high ASDRs, contributed less to the global absolute burden of LRIs due to their smaller population sizes.

#### 3.1.3. Health inequality trends

**Figure 3.**
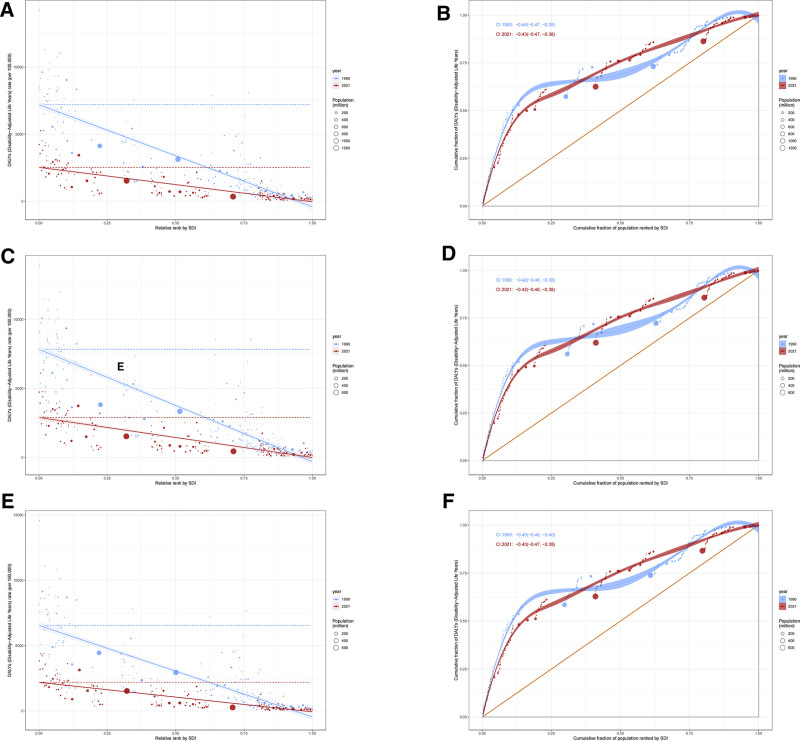
Analysis of health inequalities in the burden of LRIs across different SDI levels from 1990–2021. (A) Both SII. (B) Both CI. (C) Male SII. (D) Male CI. (E) Female SII. (F) Female CI. CI = concentration index, LRIs = lower respiratory infections, SDI = socio-demographic index, SII = slope index of inequality.

From 1990 to 2021, absolute inequality improved significantly, as evidenced by a decline in the SII from −7608.67 (95% CI: −8139.37, −7077.96) to −2560.53 (95% CI: −2815.42, −2305.63), suggesting a substantial reduction in health disparities. This improvement was more pronounced among males than females (Fig. 3A, C, and E). Nevertheless, low-SDI countries continued to experience a disproportionately higher BOD. Relative inequality also improved, albeit marginally, with the CI shifting from −0.44 (95% CI: −0.47, −0.39) to −0.43 (95% CI: −0.47, −0.38) (Fig. 3B).

### 3.2. Analysis of LRIs burden inequality, 1990 to 2021

#### 3.2.1. National and regional differences

**Figure 4.**
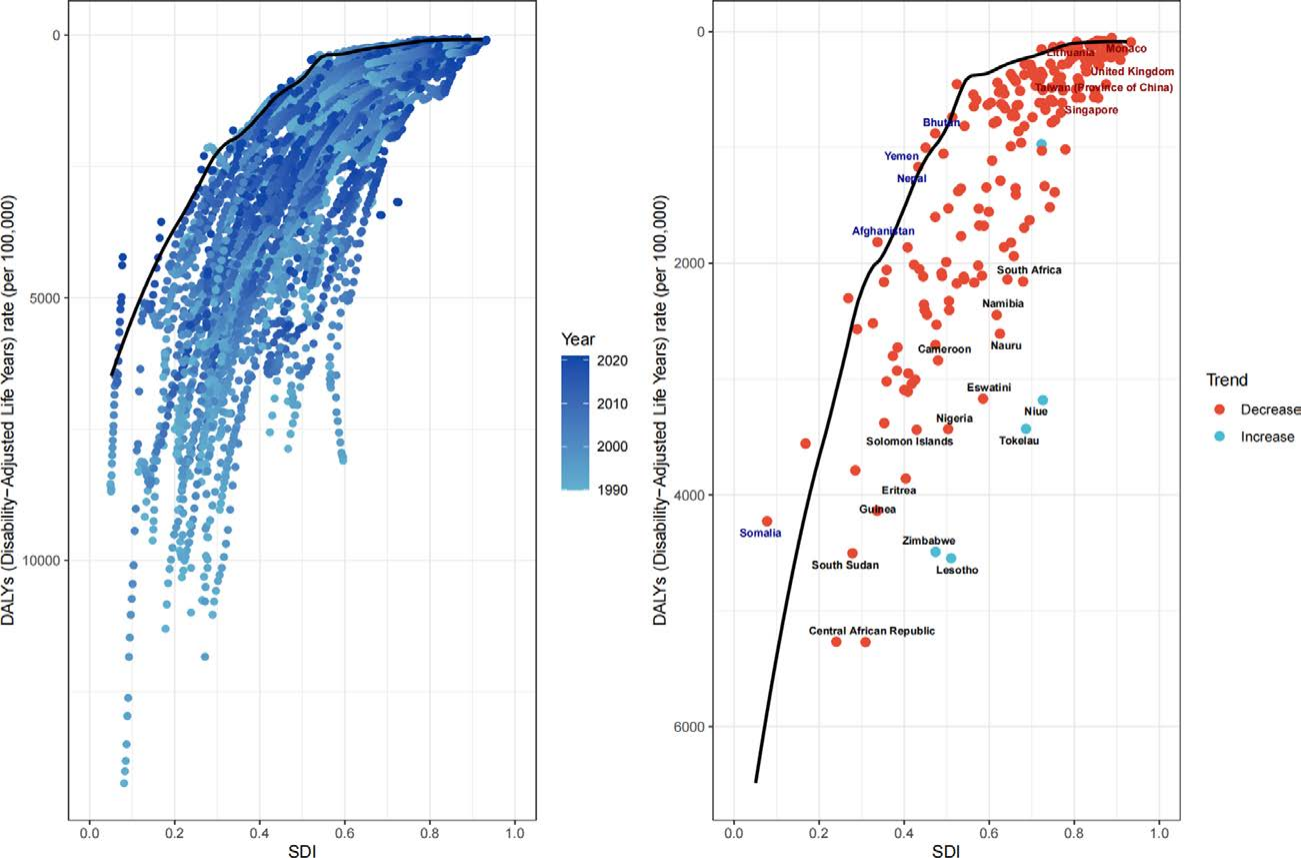
Frontier analysis of LRI burden across different countries and regions (1990–2021). LRI = lower respiratory infection.

Frontier analysis revealed a consistent reduction in LRI burden disparities with increasing SDI (Fig. 4). Despite limited resources, several low- and low-middle-SDI countries – including the Central African Republic, Eswatini, Nigeria, Eritrea, Nauru, South Sudan, Solomon Islands, Namibia, Guinea, and South Africa – demonstrated significant progress in reducing LRI burden. In contrast, countries such as Lesotho, Zimbabwe, Tokelau, and Niue exhibited substantial gaps between observed and potential burden reduction, highlighting persistent challenges in these regions.

#### 3.2.2. Aging, population growth, and epidemiological differences

**Figure 5.**
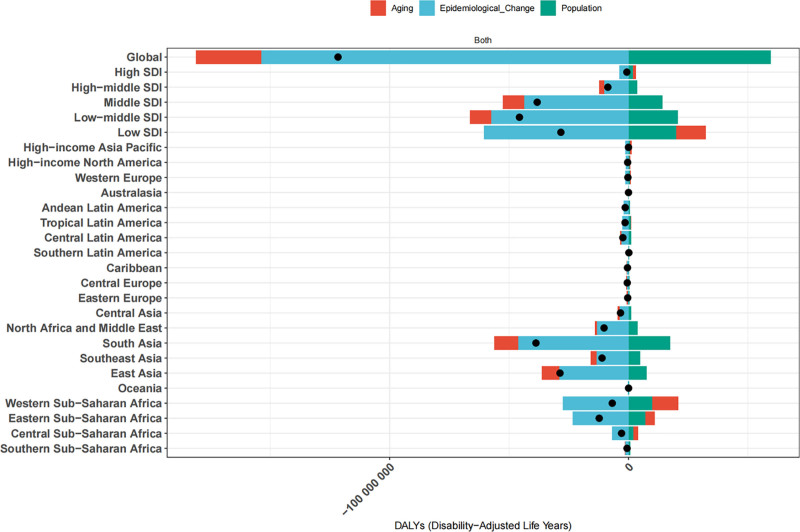
Decomposition analysis of LRI burden across different regions and SDI levels (1990–2021). LRI = lower respiratory infection, SDI = socio-demographic index.

Decomposition analysis identified epidemiological changes, particularly improved prevention and treatment strategies, as the primary drivers of global LRI burden reduction. While population growth and aging contributed to an increased burden, their impact was comparatively smaller (Fig. 5). Similar patterns were observed across both high-SDI and low-SDI countries, with epidemiological improvements significantly mitigating the adverse effects of aging and population growth. In other economic regions, epidemiological factors and aging served as protective factors, whereas population growth continued to exert a substantial impact, particularly in Asia and sub-Saharan Africa.

#### 3.2.3. Temporal trends

**Figure 6.**
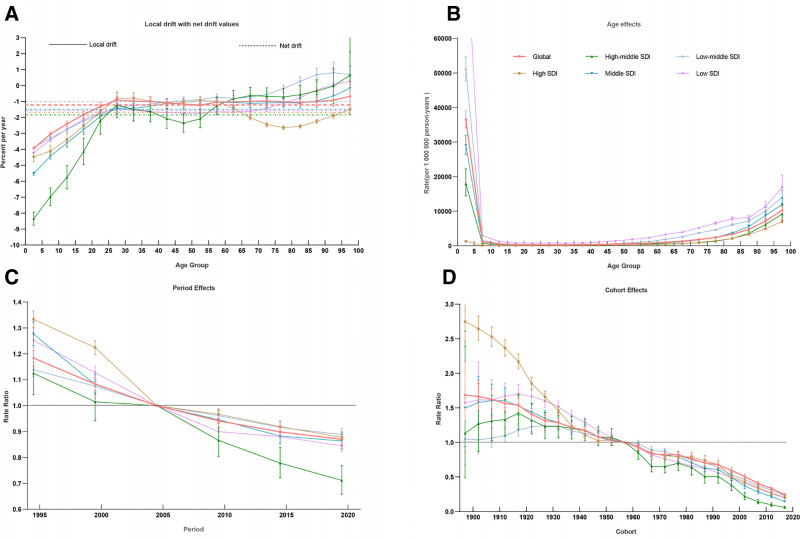
Age-period cohort distribution of ASDR in global and SDI levels, 1990–2021. ASDR = age-standardized DALY rate, DALY = disability-adjusted life year, SDI = socio-demographic index.

APC analysis revealed a net downward trend in the global burden of LRIs, with an annual decline of −1.22% (95% CI: −1.32%, −1.13%). This decline was more pronounced among males (Fig. 6A). High-middle-SDI countries experienced the largest reduction, with an annual decline of −1.82% (95% CI: −2.10%, −1.54%).

##### Age effect

Globally, the age effect exhibited a consistent pattern, with the magnitude of change directly correlated with SDI levels. The age effect declined rapidly to its lowest point and then gradually increased with age, peaking among children under 5 years old and adults over 65 years old (Fig. 6B).

##### Period effect

The global period effect coefficient demonstrated a continuous decline, strongly correlated with trends in economic development. Substantial reductions were observed before 2005, primarily driven by high-SDI countries, followed by a stabilization phase with ongoing declines in middle-high-SDI countries (Fig. 6C).

##### Cohort effect

Compared to the reference cohort (1957–1962), the risk of LRI infection decreased globally across successive birth cohorts. For early birth cohorts (before 1947), changes were predominantly influenced by high-SDI regions, with a RR reduction of 1.74 to 2.74 over 50 years. Over the past 3 decades (since 1987), the most significant improvements were observed in middle-high-SDI and middle-SDI regions. In low-middle-SDI and low-SDI regions, LRI risk initially increased before declining, peaking around 1922 (Fig. 6D).

## 4. Discussion

This study systematically analyzed the current status and determinants of health inequalities in the burden of LRIs. The findings indicate a significant decline in both the absolute DALYs and ASDRs. However, the absolute burden of LRIs remains heavily influenced by population size. In 2021, the top 5 countries with the highest absolute burden were India, Nigeria, China, Pakistan, and Ethiopia, while Australia had the lowest DALY burden and ASDR. At the national level, African countries bear the heaviest burden, whereas China demonstrated the most substantial improvement. Regionally, low-SDI countries continue to exhibit a higher burden. These findings underscore the need for targeted efforts to address the underlying drivers of inequality and reduce the population-level burden of LRIs.

The burden of LRIs is significantly influenced by regional factors. In Asia and sub-Saharan Africa, population growth has contributed substantially to the health burden. At the national level, China’s remarkable progress may be attributed to comprehensive national strategies and societal improvements. The Chinese government has implemented national development programs for public health.^[12,13]^ Additionally, Chinese researchers have extensively studied the impact of socio–demographic factors and climate change on LRIs.^[14]^ Economic growth, improved healthcare systems, and better nutrition have also played critical roles.^[15]^ However, due to its large population,^[16]^ LRIs remain a significant public health challenge in China, necessitating continued efforts in maternal and child health, tobacco and alcohol control, and air quality improvement.^[17]^

The reduction in LRI burden has been largely driven by epidemiological changes, particularly advancements in medical treatments and vaccine distribution. High-SDI countries have seen the most significant improvements, likely due to their robust healthcare systems and high-quality medical services. For instance, the introduction of PCR diagnostics for “Bordetella pertussis” has increased detection rates by 8% since the expanded program on immunization began in 1974.^[18]^ However, vaccine equity remains a challenge,^[19]^ particularly in LMICs, where disparities in wealth, education, and geography hinder vaccination rates.^[20]^ While China has achieved high vaccination coverage in most provinces,^[21]^ sub-Saharan Africa continues to struggle, with a vaccination acceptance rate of only 38.7% among adolescents and youths.^[22]^ Tailored health promotion strategies are needed to encourage vaccination in these regions.

Population growth, combined with lifestyle factors, also influences LRI burden, particularly through housing conditions, nutrition, and access to clean water. These risks are more prevalent in low-SDI countries and among indigenous populations. For example, overcrowded housing increases LRI risk by 1.69 times among indigenous Australians.^[23]^ Indoor air pollution from solid fuels is a significant risk factor for childhood pneumonia in Asia and Africa.^[24]^ Malnutrition, common in LMICs, exacerbates LRI incidence and mortality, highlighting the need to prioritize nutritional interventions.^[25]^ Handwashing, a low-cost intervention, can reduce acute respiratory infections by 16%,^[26,27]^ yet access to water and soap remains limited in many developing regions.^[28]^ In sub-Saharan Africa, 66.16% of households lack adequate handwashing facilities,^[29]^ underscoring the need for systemic investments to improve infrastructure and awareness. Regular physical activity has also been shown to reduce pneumonia risk and mortality.^[30]^

Age-related inequalities highlight the vulnerability of children under 5 and adults over 65. In 2013, the WHO and UNICEF launched the “Global Action Plan for the prevention and control of pneumonia and Diarrhoea (GAPPD)”, aiming to eliminate preventable child deaths from pneumonia and diarrhea by 2025. While global pediatric LRI mortality has declined due to vaccination and public health measures, children in low- and low-middle-SDI countries continue to bear a disproportionate burden. Similarly, as global populations age, reducing LRI burden among older adults has become a critical priority.^[31]^ However, research on the economic and systemic impacts of LRIs in older adults is largely limited to high-income countries,^[32]^ leaving significant gaps in data from LMICs, particularly in Southeast Asia and Africa. Addressing these gaps is essential to mitigate the economic and social burdens of LRIs in aging populations.

## 5. Study limitations

Limitations of this study include the availability and quality of data, particularly in low-income settings, which may affect the accuracy of burden estimates. Additionally, this study did not differentiate between LRI burden attributable to specific pathogens, an area warranting further investigation.

## 6. Conclusion

The disease burden of LRIs is deeply intertwined with health inequities, necessitating multifaceted interventions – such as equitable vaccine distribution, environmental regulation, and precision medicine – to mitigate disparities across regions and populations. Concurrently, enhanced long-term health surveillance for vulnerable groups is imperative.^[33,34]^

Further research should prioritize elucidating the LRI burden among individuals aged >5 years, particularly the association between recurrent infections and long-term pulmonary dysfunction, as well as the synergistic mechanisms of air pollution and pathogen co-exposure in exacerbating LRIs.^[35,36]^ Equitable vaccine allocation, antibiotic stewardship, and primary healthcare capacity building must be integrated into the global health agenda to establish a tiered prevention framework targeting LRIs.

## Acknowledgments

We acknowledge the contributions of the 2021 Global Burden of Disease Study collaborators.

## Author contributions

**Conceptualization:** Qing-Qing Jiang.

**Data curation:** Qing-Qing Jiang, Xiao-Yu Zhang, Xiao Yu.

**Formal analysis:** Qing-Qing Jiang.

**Investigation:** Qing-Qing Jiang.

**Software:** Xiao-Yu Zhang.

**Supervision:** Xiao-Yu Zhang, You-De Liu, Wei Pan, Jian Xue.

**Validation:** Jian Xue.

**Visualization:** Qing-Qing Jiang, Xiao Yu, You-De Liu.

**Writing – original draft:** Qing-Qing Jiang.

**Writing – review & editing:** Xiao-Yu Zhang, Xiao Yu, You-De Liu, Wei Pan, Jian Xue.
